# Quantitative normal values of helical flow, flow jets and wall shear stress of healthy volunteers in the ascending aorta

**DOI:** 10.1007/s00330-022-08866-5

**Published:** 2022-05-25

**Authors:** Sebastian Ebel, Alexander Kühn, Abhinav Aggarwal, Benjamin Köhler, Benjamin Behrendt, Robin Gohmann, Boris Riekena, Christian Lücke, Juliane Ziegert, Charlotte Vogtmann, Bernhard Preim, Siegfried Kropf, Bernd Jung, Timm Denecke, Matthias Grothoff, Matthias Gutberlet

**Affiliations:** 1 Department of Diagnostic and Interventional Radiology, University of Leipzig – Heart Centre, Leipzig, Germany; 2 Department of Diagnostic and Interventional Radiology, University of Leipzig, Liebigstr. 20, 04103 Leipzig, Germany; 3 Department of Radiology, Mata Chanan Devi Hospital of New Delhi, New Delhi, India; 4 Department of Simulation and Graphics, University of Magdeburg, Magdeburg, Germany; 5 Department for Biometry and Medical Informatics, University of Magdeburg, Magdeburg, Germany; 6 Department of Diagnostic, Interventional and Paediatric Radiology, University of Bern, Bern, Switzerland

**Keywords:** 4D flow, Flow patterns, Helical flow, Aortic flow, Magnetic resonance imaging

## Abstract

**Objectives:**

4D flow MRI enables quantitative assessment of helical flow. We sought to generate normal values and elucidate changes of helical flow (duration, volume, length, velocities and rotational direction) and flow jet (displacement, flow angle) as well as wall shear stress (WSS).

**Methods:**

We assessed the *temporal helical existence* (*TH*_*EX*_), *maximum helical volume* (*HV*_*max*_), *accumulated helical volume* (*HV*_*acc*_), *accumulated helical volume length* (*HVL*_*acc*_), *maximum forward velocity* (*maxV*_*for*_), *maximum circumferential velocity* (*maxV*_*circ*_), *rotational direction* (*RD*) and maximum *wall shear stress* (*WSS*) as reported elsewhere using the software tool Bloodline in 86 healthy volunteers (46 females, mean age 41 ± 13 years).

**Results:**

*WSS* decreased by 42.1% and *maxV*_*for*_ by 55.7% across age. There was no link between age and gender regarding the other parameters.

**Conclusion:**

This study provides age-dependent normal values regarding *WSS* and *maxV*_*for*_ and age- and gender-independent normal values regarding *TH*_*EX*_, *HV*_*max*_, *HV*_*acc*_, *HVL*_*acc*_, *RD* and _*max*_*V*_*circ*_.

**Key Points:**

*• 4D flow provides numerous new parameters; therefore, normal values are mandatory.*

*• Wall shear stress decreases over age.*

*• Maximum helical forward velocity decreases over age.*

## Introduction

The aortic diameter is not a good predictor of complications of aortic dilatation [1, 2]. The reason for using it is the law of Laplace, which links the expansion rate with the diameter of a vessel, but the biological genesis of aortic dilatation and dissection is more complex [3]. Other sources underlined that aortic flow abnormalities can contribute to the progression of dilatation [4, 5], e.g. in the setting of bicuspid aortic valve (BAV) [6–8].

4D flow allows the visualization and measurements of flow parameters like helical flow or analysis of the flow jet and may provide deeper insights into cardiovascular pathologies [9]. 4D flow might deliver new imaging biomarkers for surveillance of patients with aortic pathologies and may help to identify patients at risk for complication of aortic dilatation such as rupture and dissection, and finally to adapt patient management. This study is an extension of previous works, which have shown that absolute quantification of helical flow patterns, e.g. of helical volumes and helical duration, can help to differentiate between physiological and pathological flow patterns [10].

However, those new techniques are still neither an established part of the evaluation of patients with aortic disease, nor part of the decision-making process. Nevertheless, before it comes to analysis of pathologies, it is necessary to identify the normal range of these parameters. Thus, the aim of this study was to utilize 4D flow in the aorta of healthy individuals to generate normal values and elucidate changes of helical flow and flow jet as well as wall shear stress (WSS).

## Material and methods

### Study cohort

Eighty-six healthy volunteers (46 females, 41 ± 13 years) were included. To investigate the influence of age, we included various age groups: 19–30 (*n* = 20); 31–40 (*n* = 21); 41–50 (*n* = 21); 51–60 (*n* = 18) and ≥ 61 years (*n* = 6). The local ethics board approved the study and written informed consent was obtained from all participants.

### Magnetic resonance image acquisition

4D flow datasets were acquired at 3 T using a 16-channel surface-coil in combination with a 12-element spine-coil (Magnetom Verio Dot, Siemens Healthcare). The used 4D flow kt-GRAPPA5 sequence was validated before [11, 12]. Imaging parameters were as follows: TR = 4.6 ms, TE = 2.8 ms, Flip angle 10°, FOV 320 × 240 mm with a mean temporal resolution of 39.2ms and a spatial resolution of 2.5 × 2.5 × 2.5 mm, VENC 150 cm/s.

### Data analysis

#### Vessel segmentation, blood flow visualization and pre-processing

All processing steps were carried out using the software Bloodline (University of Magdeburg, Germany) [13–16]. The ascending aorta (ascA) was defined as the volume of the aorta between the aortic valve and the origin of the brachiocephalic trunk and the thoracic aorta as the volume between the aortic valve and the diaphragm.

Bloodline enables the analysis of defined regions, e.g. the ascA, and provides the volume as well as the length of the segmented vessel and visualizes blood flow using time-resolved pathlines [17]. We corrected for phase wraps, eddy currents and background noise as described previously [18, 19].

#### Measurements and flow quantifications

The automatic identification of complex flow patterns utilizes a recently described relative pressure calculation [10, 20]. Those parameters were evaluated recently [10]. Additionally, bloodline enables a fully automated, adaptive extraction of time-resolved flow jet information [16] and uses filtering techniques to extract the pathlines within the regions of helical flow to visualize them separately from the pathlines with non-helical flow as described by Köhler et al [17].

The parameter ***t****emporal* ***h****elical* ***ex****istence* (***TH***_***Ex***_) was introduced earlier [10]. This parameter describes the duration of helical flow throughout the cardiac cycle. This parameter was given as an absolute value in milliseconds (ms) and as a relative value in percentage (%) of the cardiac cycle.

**Fig. 1:**
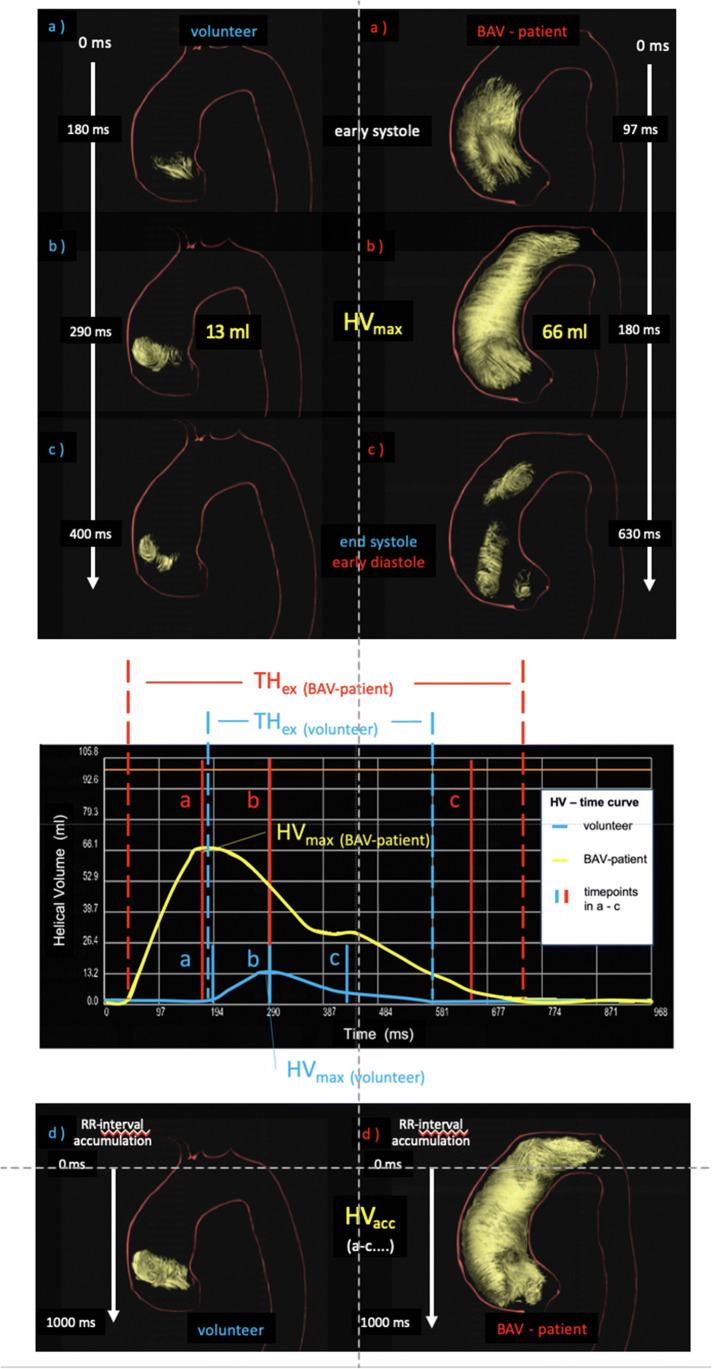
3D visualization of the evolution of a helix in the ascending aorta during the entire cardiac cycle of a healthy individual (left column) and a patient with BAV (female, 39 years old, right column). (**a**) At early systole, the helix occupies only the very proximal part of the ascending aorta in die healthy volunteer and a big part in the patient. (**b**) At mid-systole, it has grown in volume and length and reached its maximum—the ***max****imum* ***h****elical* ***v****olume* (***HV***_***max***_). (**c**) At end-systole at 300 ms, most of the helix vanished. (**d**) The summation of all volumes that contributed to the helix during the entire cardiac cycle is the ***acc****umulated* ***h****elical* ***v****olume* (***HV***_***acc***_), and its length is the ***acc****umulated* ***h****elical* ***v****olume* ***l****ength* (***HVL***_***acc***_)

The size and length of a helix were assessed with different parameters, introduced earlier [10]:

1. The ***max****imum* ***h****elical* ***v****olume* (***HV***_***max***_) as an absolute value in millilitres (ml) and the relative value in percent (%) of the volume of the thoracic aorta and of the ascA at a definite timepoint of the cardiac cycle (Fig. 1 a–c).
2. Furthermore, the ***acc****umulated* ***h****elical* ***v****olume* (***HV***_***acc***_) was defined as a summation of all volumes that contributed to the helix during the cardiac cycle. This parameter can be described as a roadmap of the helix. It is given as an absolute value in milliliters (ml) or relatively in percent (%) based on the volume of the ascA (Fig. 1 d).
3. According to the ***HV***_***acc***_, we assessed the accumulated length of a helix with the ***acc****umulated* ***h****elical* ***v****olume* ***l****ength* (***HVL***_***acc***_). This parameter describes the length of the roadmap of a helix. It is given as an absolute value in millimetres (mm) or relatively in percent (%) based on the length of the ascA (Fig. 1 d).

**Fig. 2:**
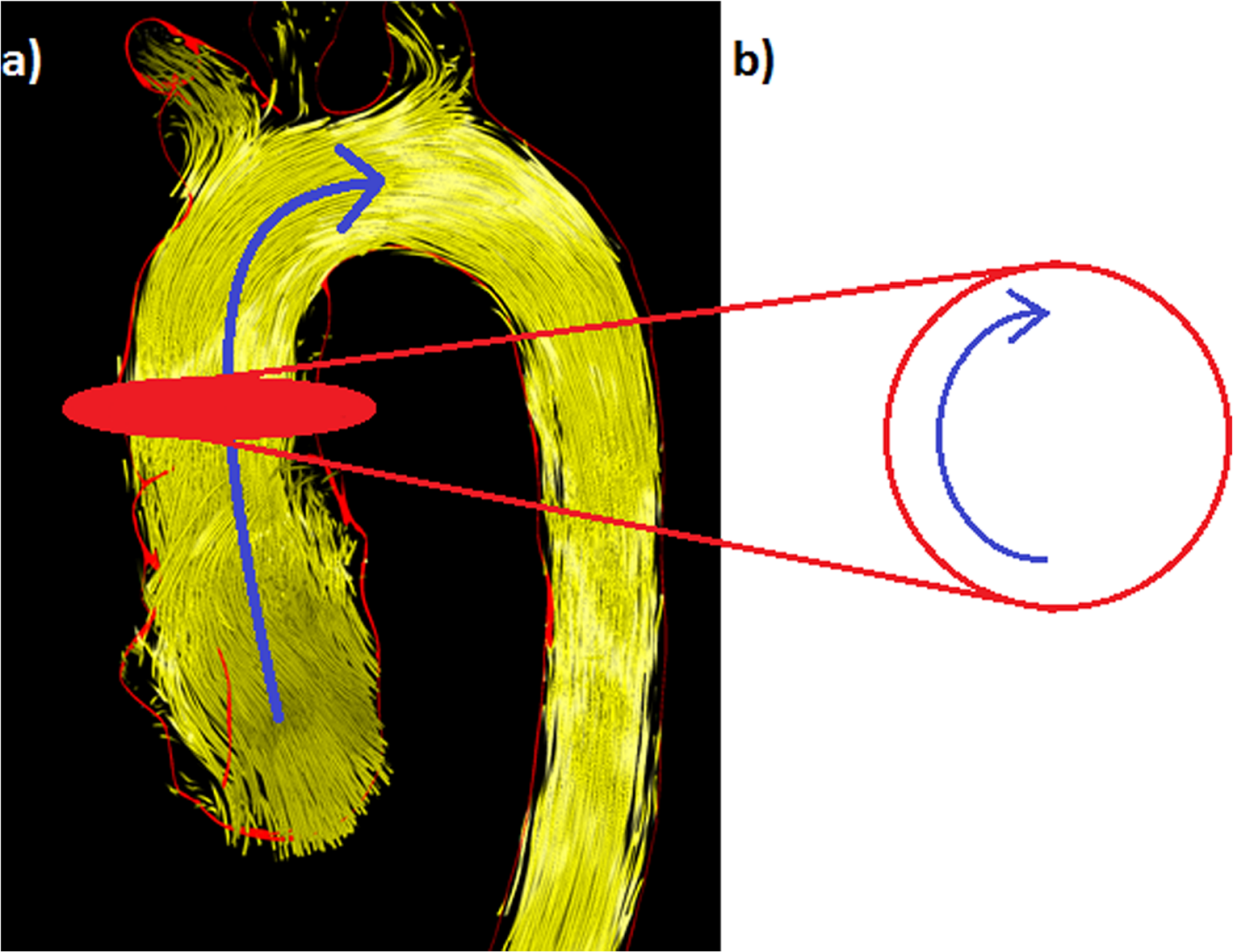
Visualization of measurements of helical velocities. (**a**) *The* ***max****imum* ***for****ward* ***v****elocity* **(maxV**_**for**_**)** in metres per second (m/s) describes the maximum helix velocity in downstream direction (blue arrow). (**b**) *The* ***max****imum* ***circ****umferential* ***v****elocity* **(maxV**_**circ**_**)** in m/s describes the maximum cross-sectional helix velocity in circumferential direction within the axial measuring plane (blue arrow)

Helical flow velocities were assessed as follows:

1. The ***max****imum* ***for****ward* ***v****elocity* (***maxV***_***for***_) of helices in metres per second (m/s) describes the maximum helix velocity in downstream direction (Fig. 2 a).
2. The ***max****imum* ***circ****umferential* ***v****elocity* (***maxV***_***circ***_) of helices in m/s describes the maximum cross-sectional helix velocity in circumferential direction (Fig. 2 b).
3. General forward flow velocity of helical and non-helical flows as a “2D parameter” in the ascA in m/s.

The *rotational direction* (*RD*) was subdivided into left-handed (*RD-*) and right-handed (*RD+*) as described by Meuschke et al [20, 21].

The flow jet was defined as suggested by Köhler et al: Aortic flow can be assumed as laminar. It follows the vessel course and the highest velocities—*the flow jet*—is usually located in the vessel’s centre; therefore, the flow jet represents the area with the highest velocities within the *parabolic flow profile* [16]. Furthermore, we evaluated the angle between the vessel’s centreline and the flow jet—*the* ***f****low* ***j****et* ***a****ngle* (***FJA***) [5] in the ascA.

Additionally, we assessed the normalized ***f****low* ***d****isplacement* (***FD***) in peak systole in the ascA as a relative, cross-sectional parameter. It is the relative distance between the fastest pixel in peak systole and the centreline normalized to the vessel radius, where 0% means that the fastest pixel lies on the vessel’s centreline and 100% means that the fastest pixel was located at the vessel wall [22].

**Fig. 3:**
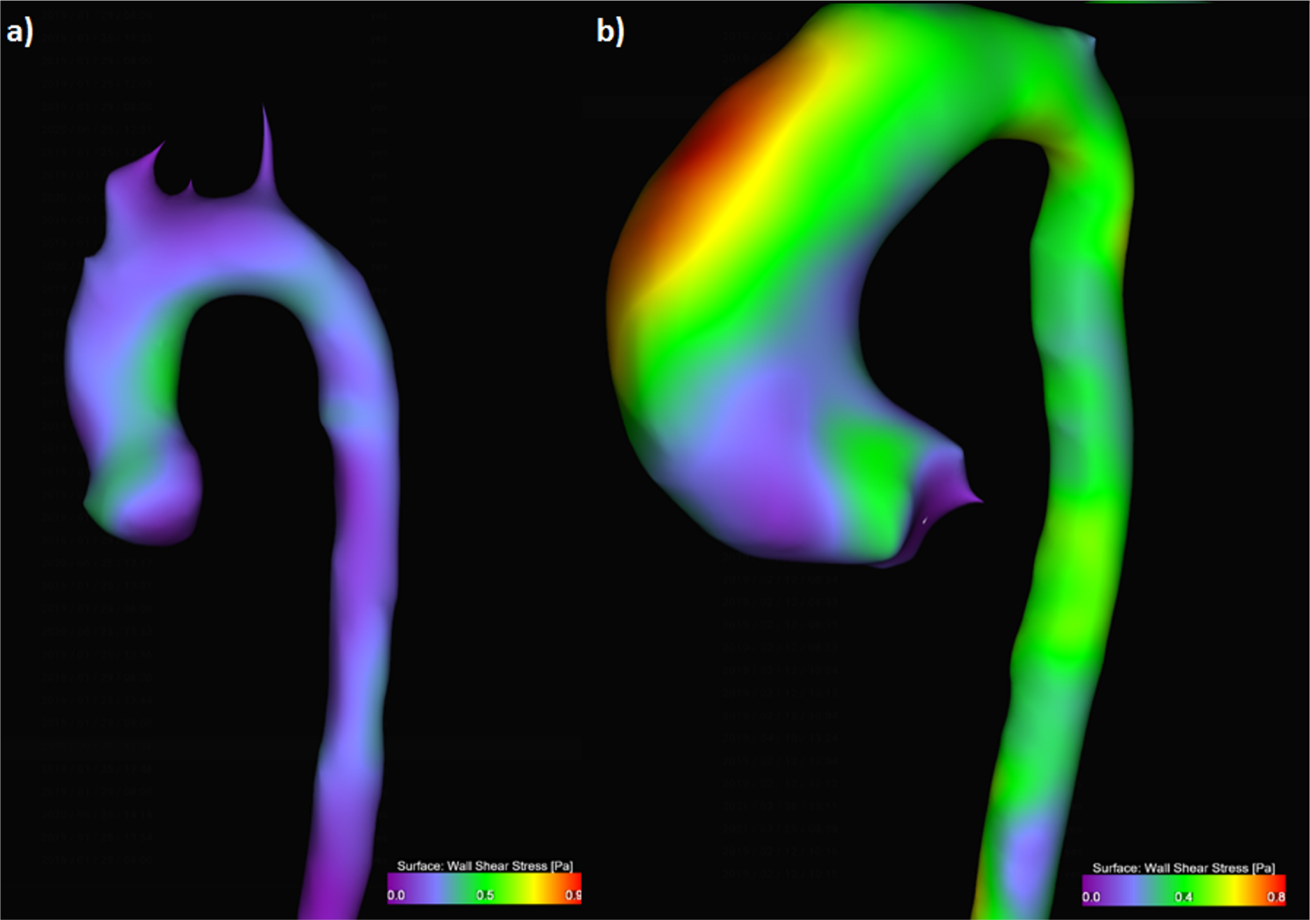
3D visualization of wall shear stress (WSS) as a heatmap. (**a**) Heatmap of a healthy volunteer normal WSS distribution of max. 0.5 Pa. (**b**) WSS heatmap of a patient with BAV with elevated WSS at the outer surface of the aortic arch of max. 0.8 Pa

Furthermore, we assessed the maximum ***w****all* ***s****hear* ***s****tress* (***WSS***_***max***_) within the ascA as an absolute value in pascal (Pa) as reported previously [23] (Fig. 3).

### Statistical analysis

All results were given as their median values and quartiles (1^st^/3^rd^). Statistical analysis was performed using the statistical software package SAS 9.4 (SAS Institute Inc.). Binary outcome variables were investigated in logistic regression analyses with age and gender as potential regressors. For the metrical outcome variables, the dependence of the target variables on age and gender or the interaction of both was checked in type II regression models using the procedure SAS/TRANSREG that automatically includes a Box-Cox transformation of the target variables to obtain approximately normal distributions of the residuals. As there were no significant dependencies on the interaction of age and gender, only the *p*-values of the tests for the main effects of age and gender are reported here. If the Box-Cox transformed target variable did not show significant dependencies on age in the corresponding two-sided linear model tests at an error level of 5%, then reference ranges for the transformed variables were derived as (mean ± 1.96 · standard deviation) where mean and standard deviation were obtained from the total sample or separately by gender depending on the significance status for the influence of gender in the above regression model. In case of age dependencies, age-dependent reference ranges were obtained as (predicted value ± 1.96 × SQRT (residual variance)), where the regression parameters were taken from the above regression model with two regressors if the influence of gender was significant, too, or from a reduced regression model with only age as regressor otherwise. Finally, the mean and the limits of these reference ranges were reverse transformed with the inverse Box-Cox transformation yielding estimates for the median and the limits of the reference range of the original variables. Variables with a range including negative values were shifted before the above-described procedure with a corresponding back shift of the reference limits and the centre at the end. The obtained reference ranges should cover approximately 95% of the healthy population.

## Results

### Volunteer characteristics

Eighty-six volunteers were included (46 females, mean age 41 ± 13 years). Mean body mass index was 24.1 ± 5.2 kg/m^2^ with no significant differences between the age groups. The mean resting heart rate during the examination was 69 ± 16 beats per minute, the mean cardiac output was 4.4 ± 1.2 l/min.

**Fig. 4:**
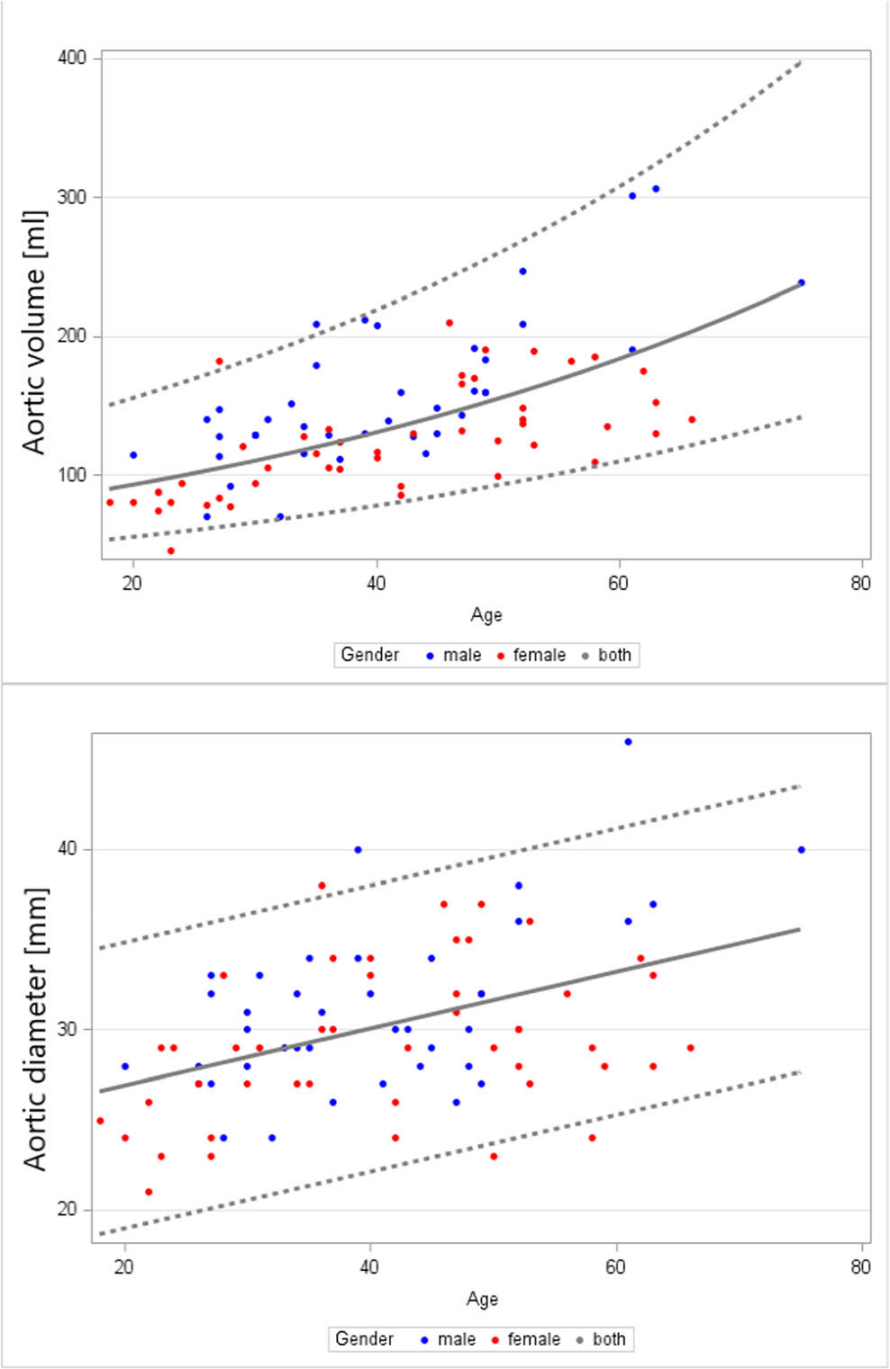
Relationship between age and geometric aortic parameters. Top: ascending aortic volume (ml), bottom: ascending aortic diameter (mm), both show a strong trend to increase with age. The grey dotted lines mark the 2.5% and 97.5% percentiles; the grey solid lines mark the median

We found a good positive correlation between age and aortic volume (*R* = 0.629, *p* = 0.002) and aortic diameter (*R* = 0.637, *p* = 0.009), indicating a steady growth of ascA with increasing age (Fig. 4 and Table 1). The correlation coefficient *R* between the aortic volume and gender was 0.406 (*p* = 0.003), revealing a bigger aorta in men.

**Table 1:** Distribution of age-dependent (***R* = 0.629** and **0.406**) and gender-independent normal ranges regarding the thoracic aortic volume and the ascending aortic diameter

Parameter	Age dependency	Gender dependency	Age	Median	Lower limit	Upper limit
Thoracic aortic volume (ml)	**Yes** **(*R* = 0.629,** ***p* = 0.002)**	No	19–30	101.2	60.4	169.6
			31–40	125.1	74.6	209.6
			41–50	148.4	88.5	248.6
			51–60	176.0	105.0	294.9
			> 60	183.5	109.5	307.4
Ascending aortic diameter (mm)	**Yes** **(*R* = 0.637,** ***p* = 0.009)**	No	19–30	24.5	19.6	33.4
			31–40	29.7	21.7	37.6
			41–50	31.2	23.3	39.2
			51–60	32.8	24.9	40.7
			> 60	33.2	25.3	41.1

The median length of the thoracic aorta was 390mm (260/420). There was no significant correlation between the aortic length and age (*R* = 0.101, *p* = 0.353) or between aortic length and gender (*R* = 0.047, *p* = 0.777).

### Presence of helices in the ascending aorta

**Fig. 5:**
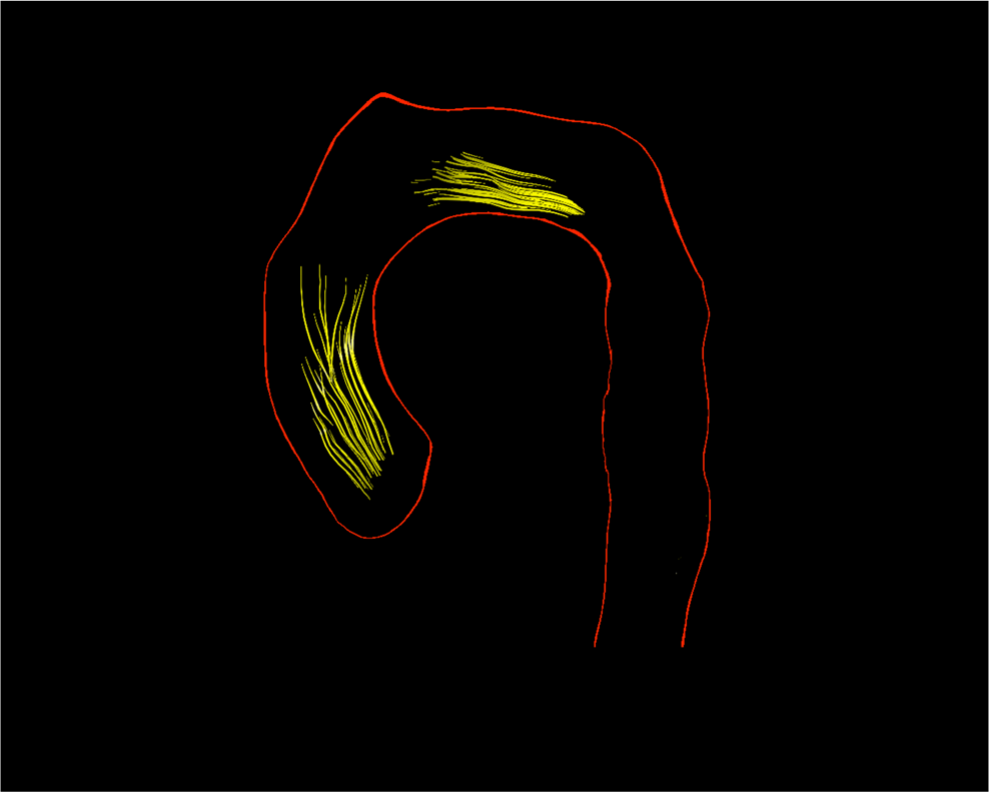
3D visualization of helices in the ascending aorta and in the aortic arch. Note the gap between both helices

We detected at least one helix in all 86 participants, only one within the ascA in 70/86 (81%) subjects. The helix started right downstream from the aortic valve and evolved through the ascA towards the aortic arch. Helices in the early systole were small (Fig. 1a), while they reach their maximum volume and length around mid-systole (Fig. 1b). In 16/86 (19%) participants, we found an additional helix in the distal ascA or the aortic arch: 7 female, *N* = 3 19–30 years, *N* = 4 31–40 years, *N* = 4 41–50 years, *N* = 4 51–60 years, *N* = 1 > 60 years. Visually, the helix in the aortic arch started right where the helix in the ascA ended (Fig. 5). We found no helices in the descending aorta.

#### Duration of helical flow in the ascending aorta throughout the cardiac cycle (***TH***_***Ex***_)

The median ***TH***_***Ex***_ in all participants in the ascA was 231.1ms (145.9/321.4), which was 25.7% (18.5/33.3) of the cardiac cycle. The helices began at 80.4 ms (68.7/134.5) (6.5% (3.1/13.2)) and ended at 337.8 ms (260.0/403.4) (35.8% (25.6/44.0)), indicating that helices in healthy volunteers mainly occurred during mid-systole and vanish in early diastole. There was no strong link between ***TH***_***Ex***_ and age (*R* = 0.366) or gender (*R* = 0.259) (Table 2).

**Table 2:** Distribution of age- and gender-independent normal ranges regarding the ***t****emporal* ***h****elical* ***ex****istence* (***TH***_***E*x**_), ***TH***_***Ex***_ begin and ***TH***_***Ex***_ end

Parameter	Age dependency	Gender dependency	Median	Lower limit	Upper limit
***T****emporal* ***h****elical* ***ex****istence* (***TH***_***Ex***_) (ms)	No	No	231.1	54.6	487.6
***T****emporal* ***h****elical* ***ex****istence* (***TH***_***Ex***_) (%)	No	No	25.7	4.1	45.7
***TH***_***Ex***_ begin (ms)	No	No	80.4	21.0	297.2
***TH***_***Ex***_ begin (%)	No	No	6.5	2.5	31.6
***TH***_***Ex***_ end (ms)	No	No	337.8	137.8	721.3
***TH***_***Ex***_ end (%)	No	No	35.8	16.4	69.6

#### *Maximum* (***HV***_***max***_) and ***acc****umulated* (***HV***_***acc***_) ***h****elical****v****olumes*

The median absolute ***HV***_***max***_ was 8.4 ml (4.8/13.4), which was 6.0% (4.9/12.3) of the volume of the thoracic aorta and 14.5% (9.1/28.2) of the volume of the ascA. We found a weak correlation between the measurements and age (*R* = 0.301; *p* = 0.106) and gender (*R* = 0.308; *p* = 0.048) in both vessel sections.

The median absolute ***HV***_***acc***_ was 9.3 ml (4.8/13.4), which was 7.6% (4.9/12.3) of the volume of the thoracic aorta and 19.4% (12.1/23.1) of the volume of the ascA. Although there was a mediocre correlation between the measurements and age, it was not statistically significant (*R* = 0.401; *p* = 0.101). Additionally, there was a weak correlation between the measurements and gender (*R* = 0.040) in both vessel sections (Table 3).

**Table 3:** Distribution of age- and gender-independent normal ranges regarding the ***max****imum* ***h****elical* ***v****olume* (***HV***_***max***_), the ***acc****umulated* ***H****ELICAL* ***V****OLUMe* (***HV***_***ac*c**_) and the ***acc****umulated* ***h****elical* ***v****olume* ***l****ength* (***HVL***_***acc***_)

Parameter	Age dependency *R*	Gender dependency *R*	Median	Lower limit	Upper limit
***Max****imum* ***h****elical* ***v****olume* (***HV***_***max***_) (ml)	0.301	0.308	8.4	1.5	27.9
***HV***_***max***_ thoracic aorta (%)	0.298	0.211	6.0	0.8	13.7
***HV***_***max***_ ascending aorta (%)	0.308	0.308	14.5	2.0	35.4
***Acc****umulated* ***h****elical* ***v****olume* (***HV***_***acc***_) (ml)	0.401	0.040	9.3	1.97	39.6
***HV***_***acc***_ thoracic aorta (%)	0.389	0.015	7.6	1.88	23.7
***HV***_***acc***_ ascending aorta (%)	0.451	0.051	19.4	2.72	50.2
***Acc****umulated* ***h****elical* ***v****olume* ***l****ength* (***HVL***_***acc***_) (mm)	0.269	0.096	58	13	108
***HVL***_***acc***_ thoracic aorta (%)	0.277	0.101	16.3	4.0	34.2
***HVL***_***acc***_ ascending aorta (%)	0.274	0.084	56.6	8.0	91.7

#### ***Acc****umulated****h****elical****v****olume****l****ength* (***HVL***_***acc***_) of helices in the ascending aorta

The median absolute ***HVL***_***acc***_ overall was 58 mm (31/61), which was 16.3% (11.6/21.5) of the length of the thoracic aorta and 56.6% (32.9/75.2) of the length of the ascA. There was a weak correlation between the measurements and age (*R* = 0.269; *p* = 0.022) and no correlation for gender (*p* = 0.096; *p* = 0.573) in both vessel sections (Table 3).

#### ***For****ward* (***V***_***for***_) and ***circ****umferential* (***V***_***circ***_) ***v****elocities* of helices in the ascending aorta

**Fig. 6:**
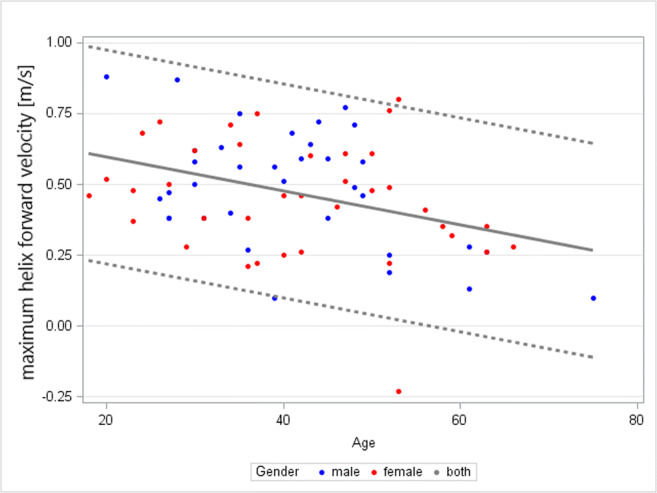
Relationship between age and the helix ***for****ward* ***v****elocity* (***V***_***for***_) (m/s), showing a strong trend to decrease with age. The grey dotted lines mark the 2.5% and 97.5% percentiles; the grey solid lines mark the median

The median maximum ***V***_***for***_ and ***V***_***circ***_ overall were 57.4 cm/s (47.5/79.2) and 70.1 cm/s (60.6/83.4). ***V***_***for***_ decreased by 55.7% across age (*R* = − 0.538) (*p* = 0.038), while there was no link between ***V***_***circ***_ and age (*R* = 0.272, *p* = 0.355) or gender (*R* = 0.211, *p* = 0.793) (Table 4 and Fig. 6). Mean “2D forward flow velocity” was 118.9 cm/s (96.2/177.1) in the ascA; it decreased by 29.8% over age (*R* = −0.383, *p* = 0.028).

**Table 4:** Distribution of age-dependent and gender-independent normal ranges regarding the maximum helical ***for****ward* ***v****elocity* (***V***_***for***_) and the age- and gender-independent maximum helical ***circ****umferential* ***v****elocity* (***V***_***circ***_)

Parameter	Age dependency	Gender dependency	Age	Median	Lower limit	Upper limit
Maximum helical ***for****ward* ***v****elocity* (***V***_***for***_) (cm/s)	**Yes** **(*R* = − 0.583,** ***p* = 0.038)**	No	19–30	58.9	20.8	96.2
			31–40	53.2	14.5	89.7
			41–50	50.0	9.0	83.6
			51–60	44.4	7.3	81.4
			> 60	38.1	4.7	80.3
Maximum helical ***circ****umferential* ***v****elocity* (***V***_***circ***_) (cm/s)	No	No	-	70.1	18.9	103.1

#### ***R****otational****d****irection* (***RD***) of helices in the ascending aorta

In 61 (71%) cases, helical flow was mainly right-handed, and in 25 cases, we found mainly left-handed helical flow. There was no significant correlation between ***RD*** and age (*R* = 0.228, *p* = 0.748 and *R* = 0.212, *p* = 0.840) or ***RD*** and gender (*R* = 0.241, *p* = 0.392 and *R* = 0.225, *p* = 0.572).

#### ***F****low****j****et****a****ngle* (***FJA***_***sys***_)

The median ***FJA***_***sys***_ was 2.3° (1.6/3.5). There was no significant link between the ***FJA*** and age (*R* = 0.322, *p* = 0.892) and the ***FJA*** and gender (*R* = 0.029, *p* = 0.697) (Table 5).

**Table 5:** Distribution of age- and gender-independent normal ranges regarding the peak ***sys****tolic* ***f****low* ***j****et* ***a****ngle* (***FJA***_***sys***_) the age-dependent and gender-independent ***max****imum* ***w****all* ***s****hear* ***s****tress* (***WSS***_***max***_), ***R* = 0.514**

Parameter	Age dependency	Gender dependency	Age	Median	Lower limit	Upper limit
Peak ***sys****tolic* ***f****low* ***j****et* ***a****ngle* (***FJA***_***sys***_) (°)	No	No	-	2.3	1.9	3.5
Maximum ***WSS*** (**WSS**) (Pa)	**Yes** **(*R* = − 0.514,** ***p* = 0.039)**	No	19–30	0.9	0.4	3.9
			31–40	0.6	0.3	2.6
			41–50	0.5	0.2	2.1
			51–60	0.5	0.2	1.7
			> 60	0.4	0.2	1.5

#### ***F****low****d****isplacement* (***FD***)

The median peak systolic normalized ***f****low* ***d****isplacement* was 1.5% (0.9/2.1) with no significant correlation between the measurements and age (*R* = 0.137, p = 0.480) or gender (= 0.011, *p* = 0.527).

#### ***W****all****s****hear****s****tress* (***WSS***)

The median maximum ***WSS*** in the ascA was 0.61Pa (0.52/1.01). There was a significant (*R* = − 0.514, *p* = 0.039) negative correlation between ***WSS*** and age, but no link between ***WSS*** and gender (*R* = 0.198, *p* = 0.320) (Table 5): From 18 to 60 years, ***WSS*** decreased by 42.1%.

## Discussion

Numerous 4D flow parameters were already described [19, 24–26]. These parameters were mostly assessed visually only, which might limit the use in the clinical routine. We are convinced that quantitative parameters, which can be assessed in a highly standardized manner, might be one way to overcome this limitation. We utilized 4D flow to establish normal values regarding helical flow, the flow jet and WSS in the ascA in a large cohort.

In line with other studies, we found that the ascA diameter increased with age [3, 27]: Our calculations regarding the normal range of the ascending aortic diameter indicate an upper limit > 40 mm in volunteers > 51 years, which could be interpreted as an aortic ectasia [1, 2]. Since we only included healthy subjects in this study, one possible conclusion—in line with other sources—could be that the aortic diameter alone is a poor parameter for the evaluation of aortic diseases [1, 3, 28].

Furthermore, we were able to detect at least one helix in the ascA in all participants. Additionally, we found a second helix in the aortic arch in 16 (19%) participants. Interestingly, the aortic arch helices started right at the spot where the ascending aortic helices ended. We state that this sharp separation of the helices is most likely artificial due to the used technique of pressure-based helix extraction. However, it shows that there seems to be a “transition-zone” in between those two helices, right at the transition from the ascA to the aortic arch. In other words: There are differences regarding pressure and flow characteristics between the ascA and the aortic arch, which is in accordance with the literature: Frydrychowicz et al found significant differences in the distribution of WSS between the ascA and the aortic arch. The authors stated that this is helpful for explaining why atherosclerotic lesions predominantly develop and progress mainly at the origins of the supra-aortic vessels [29]. Other sources underlined that the flow abnormalities in the setting of BAV might extend into the aortic arch (Fig. 1), which fits our observation [6].

We found out that in the ascA, helices mainly occur during systole and vanish during diastole. This is in line with the literature: Kilner et al [30] described “spiral” flow predominantly occurring during systole. Other studies investigated the evolution of helical flow by visual analyses and demonstrated that helical flow emerges mainly in peak systole [31].

Additionally, helices in the early systole were usually small, while they reached their maximum volume and length around mid-systole. We assume that some small helices were missed due to the overlay of pathlines with laminar and helical flow, while (semi)automatic analyses might enable the detection of even small helices.

We found a ***TH***_***EX***_ of 25.7% to be normal; previously, it has been shown that ***TH***_***EX***_ in BAV patients can be more than 2-fold elevated [10], indicating that ***TH***_***EX***_ can be helpful discriminating normal and abnormal flow.

This current study extends previous attempts to utilize the technique of pressure-based helix extraction to measure volumes and lengths of helices. We found a ***HV***_***acc***_ of up to 39.6ml to be normal, whereas a recent study found a ***HV***_***acc***_ of up to 236ml in BAV patients [10].

The helical maximum flow velocities within a helix in healthy volunteers were generally lower than the general “2D peak flow velocities”. We found a reduction of helical *forward velocity* (***V***_***for***_) by 55.7% with age (18 to 63 years), while there was no link between age or gender and the *circumferential velocity* (***V***_***circ***_), and we found a general decrease of peak flow velocity by 29.8% in the ascA. One possible explanation for this finding could be the ascA diameter increases causing a slower laminar and non-laminar flow in general. This is partially in line with the results of van Ooij et al [32]. They investigated the maximum velocity of bulk blood flow and demonstrated decreasing velocities with age. Although they did not investigate helical blood flow, their findings fit our findings, indicating decreasing forward velocities with age. Contrarily, we found no correlation between the helices’ *circumferential velocity* (***V***_***circ***_) and age or gender. To the best of our knowledge, there is no study that has systematically evaluated the impact of circumferential helical flow velocities, yet.

In line with Lorenz et al, we found both right- and left-handed helical flows to be normal in healthy volunteers, suggesting that this parameter cannot distinguish between healthy and pathological flows [33].

Dyverfeldt et al stated that the normalized *flow displacement* (***FD***) is a suitable parameter for identifying and risk-stratifying patients who are likely to develop clinically significant aortic dilation, but their study did not include healthy volunteers [34]. Later, Sigovan et al compared patients with BAV and seven healthy volunteers and found the normalized *flow displacement* (***FD***) to be of importance for distinguishing between physiological and pathological flows [35]. We found a normalized ***FD*** of 0.02 to be normal; they found a mean normalized flow displacement of 0.12 in patients while Dux-Santoy et al found a normalized displacement of 0.05–0.08 in patients with BAV [6]. This suggests that the parameter “normalized displacement” can distinguish between physiological and pathological flows. As expected, we found that *WSS* was closely related to *forward velocity*: Both parameters demonstrated a negative correlation with age. van Ooij et al investigated *WSS* in healthy volunteers and found a decreasing *WSS* with normal aging [32, 36]. Additionally, they elucidated *WSS* in patients with BAV and found a good correlation between abnormal velocities and *WSS*. They found *WSS* of 0.8 to be normal for healthy adults < 30 years; in our study, we found 0.88 Pa to be normal for this age group (the differences between those values occur due to slightly different scan parameters).

They found significantly elevated *WSS* in BAV patients using matched WSS maps [32, 36]; therefore, *WSS* could potentially be an important marker for aortic dilatation. It is known that WSS measurements depend strongly on the used scan parameters [26, 37]. This indicates that the here provided normal values only apply when using the same parameters as reported.

One limitation of our study is that we did not differentiate between helices and vortices. A visual, qualitative but also a quantitative differentiation between both is often not possible. There is a smooth transition between both phenomena and definite cutoffs do not exist. Nevertheless, this differentiation could be of interest because there is evidence that vortical flow is a relevant factor, e.g. in aortic dissection, and should be addressed in future research [38].

In conclusion, we demonstrated in 86 healthy volunteers that strong correlations exist between age and the hemodynamic parameters helical *forward velocity* and *WSS* at peak systole. Interestingly, the “*spatio-temporal helical parameters*” like volume, length and temporal existence did not depend on gender and age. We elucidated that the parameters ***TH***_***EX***_, the helical volumes and normalized displacement enable to discriminate between physiological and pathological flows. We provided normal ranges for all these flow parameters, which might be an important presupposition for the assessment of patients with aortic disease.

## Declarations

### Guarantor

The scientific guarantor of this publication is Prof. Dr. Matthias Gutberlet.

### Statistics and biometry

One of the authors has significant statistical expertise.

### Methodology

• prospective

• diagnostic or prognostic study

• performed at one institution

## Abbreviations

4D: Four dimensional
ascA: Ascending aorta
BAV: Bicuspid aortic valve
dia: Diastolic
FJA: Flow jet angle
HV_acc_: Accumulated helical volume
HVL_acc_: Accumulated helical volume length
HV_max_: Maximum helical volume
maxV_circ_: Maximum circumferential velocity
maxV_for_: Maximum forward velocity
min: Minute
ml: Millilitre
mm: Millimetre
MRI: Magnetic resonance imaging
RD: Rotational direction
SD: Standard deviation
sys: Systolic
TH_EX_: Temporal helical existence
WSS: Wall shear stress

## References

[1] Coady MA, Rizzo JA, Hammond GL et al (1997) What is the appropriate size criterion for resection of thoracic aortic aneurysms? J Thorac Cardiovasc Surg. 10.1016/S0022-5223(97)70360-X 10.1016/S0022-5223(97)70360-X 9081092

[2] Pape LA, Tsai TT, Isselbacher EM et al (2007) Aortic diameter ≥5.5 cm is not a good predictor of type A aortic dissection: Observations from the International Registry of Acute Aortic Dissection (IRAD). Circulation. 10.1161/CIRCULATIONAHA.107.702720 10.1161/CIRCULATIONAHA.107.702720 17709637

[3] Callaghan FM, Bannon P, Barin E, Celemajer D, Jeremy R, Figtree G, Grieve SM (2018) Age-Related Changes of Shape and Flow Dynamics in Healthy Adult Aortas: A 4D Flow MRI Study. J Magn Reson Imaging:1–11. 10.1002/jmri.26210 10.1002/jmri.26210 30102443

[4] Hope MD, Hope TA, Crook SES, Ordovas KG, Urbania TH, Alley MT, Higgins CB (2011). 4D flow CMR in assessment of valve-related ascending aortic disease. JACC Cardiovasc Imaging.

[5] Den Reijer PM, Sallee D, Van Der Velden P, Zaaijer E, Parks WJ, Ramamurthy S, Robbie T, Donati G, Lamphier C, Beekman R, Brummer M (2010). Hemodynamic predictors of aortic dilatation in bicuspid aortic valve by velocity-encoded cardiovascular magnetic resonance. J Cardiovasc Magn Reson.

[6] Dux-Santoy L, Guala A, Teixidó-Turà G, Ruiz-Muñoz A, Maldonado G, Villalva N, Galian L, Valente F, Gutiérrez L, González-Alujas T, Sao-Avilés A, Johnson KM, Wieben O, Huguet M, García-Dorado D, Evangelista A, Rodríguez-Palomares JF (2019) Increased rotational flow in the proximal aortic arch is associated with its dilation in bicuspid aortic valve disease. Eur Hear J Cardiovasc Imaging 20:1407–1417. 10.1093/ehjci/jez046 10.1093/ehjci/jez046 30919887

[7] Bissell MM, Hess AT, Biasiolli L, Glaze SJ, Loudon M, Pitcher A, Davis A, Prendergast B, Markl M, Barker AJ, Neubauer S, Myerson SG (2013). Aortic dilation in bicuspid aortic valve disease: Flow pattern is a major contributor and differs with valve fusion type. Circ Cardiovasc Imaging.

[8] Rodríguez-Palomares JF, Dux-Santoy L, Guala A, Kale R, Maldonado G, Teixidó-Turà G, Galian L, Huguet M, Valente F, Gutiérrez L, González-Alujas T, Johnson KM, Wieben O, García-Dorado D, Evangelista A (2018). Aortic flow patterns and wall shear stress maps by 4D-flow cardiovascular magnetic resonance in the assessment of aortic dilatation in bicuspid aortic valve disease. J Cardiovasc Magn Reson.

[9] van der Geest RJ, Garg P (2016). Advanced Analysis Techniques for Intra-cardiac Flow Evaluation from 4D Flow MRI. Curr Radiol Rep.

[10] Ebel S, Josefin D, Köhler B, Preim B, Benjamin B, Riekena B, Bernd J, Stehning C, Kropf S, Grotho M, Gutberlet M (2020) Automated Quantitative Extraction and Analysis of 4D flow Patterns in the Ascending Aorta : An intraindividual comparison at 1 . 5 T and 3 T. Sci Rep:1–13. 10.1038/s41598-020-59826-2 10.1038/s41598-020-59826-2 PMC7031260 32076060

[11] Ebel S, Dufke J, Köhler B, Preim B, Rosemeier S, Jung B, Dähnert I, Lurz P, Borger M, Grothoff M, Gutberlet M (2019). Comparison of two accelerated 4D-flow sequences for aortic flow quantification. Sci Rep.

[12] Ebel S, Hübner L, Köhler B, Kropf S, Preim B, Jung B, Grothoff M, Gutberlet M (2019) Validation of two accelerated 4D flow MRI sequences at 3 T : a phantom study. Eur Radiol Exp 3:12 10.1186/s41747-019-0089-2 PMC6391502 30806827

[13] Köhler B, Grothoff M, Gutberlet M, Preim B (2019) Bloodline: A system for the guided analysis of cardiac 4D PC-MRI data. Comput Graph 82:32–43

[14] Koehler B, Gasteiger R, Preim U, Theisel H, Gutberlet M, Preim B (2013). Semi-automatic vortex extraction in 4D PC-MRI cardiac blood flow data using line predicates. IEEE Trans Vis Comput Graph.

[15] Köhler B, Preim U, Grothoff M, Gutberlet M, Fischbach K, Preim B (2015) Guided Analysis of Cardiac 4D PC-MRI Blood Flow Data. Eurographics (Dirk Bartz Prize) 2015 2–5

[16] Köhler B, Grothoff M, Gutberlet M, Preim B (2018). Visual and quantitative analysis of great arteries’ blood flow jets in cardiac 4D PC-MRI data. Comput Graph Forum.

[17] Köhler B, Grothoff M, Gutberlet M, Preim B (2019) Computers & Graphics Bloodline : A system for the guided analysis of cardiac 4D PC-MRI data [DRAFT]. Comput Graph

[18] Bock J, Kreher BW, Hennig J, Markl M (2007) Optimized pre-processing of time-resolved 2D and 3D Phase Contrast MRI data. Proc 15th Annu Meet ISMRM 15:3138

[19] Dyverfeldt P, Bissell M, Barker AJ, Bolger AF, Carlhäll CJ, Ebbers T, Francios CJ, Frydrychowicz A, Geiger J, Giese D, Hope MD, Kilner PJ, Kozerke S, Myerson S, Neubauer S, Wieben O, Markl M (2015). 4D flow cardiovascular magnetic resonance consensus statement. J Cardiovasc Magn Reson.

[20] Köhler B, Grothoff M, Gutberlet M, Preim B (2018) Pressure-based vortex extraction in cardiac 4D PC-MRI blood flow data. EUROVIS 0–4. 10.2312/eurovisshort.20181071

[21] Meuschke M, Köhler B, Preim U, Preim B, Lawonn K (2016). Semi-automatic Vortex Flow Classification in 4D PC-MRI Data of the Aorta. Comput Graph Forum.

[22] Sigovan M, Hope MD, Dyverfeldt P, Saloner D (2011). Comparison of four-dimensional flow parameters for quantification of flow eccentricity in the ascending aorta. J Magn Reson Imaging.

[23] Potters WV, Marquering HA, VanBavel E, Nederveen AJ (2014). Measuring Wall Shear Stress Using Velocity-Encoded MRI. Curr Cardiovasc Imaging Rep.

[24] Markl M, Frydrychowicz A, Kozerke S, Hope M, Wieben O (2012). 4D flow MRI. J Magn Reson Imaging.

[25] Stankovic Z, Allen BD, Garcia J, Jarvis KB, Markl M (2014). 4D flow imaging with MRI. Cardiovasc Diagn Ther.

[26] Stalder AF, Russe MF, Frydrychowicz A, Bock J, Hennig J, Markl M (2008). Quantitative 2D and 3D phase contrast MRI: Optimized analysis of blood flow and vessel wall parameters. Magn Reson Med.

[27] Rogers WJ, Hu Y, Coast D, Vido DA, Kramer CM, Pyeritz RE, Reichek N (2001). Age-Associated Changes in Regional Aortic Pulse Wave Velocity. J Am Coll Cardiol.

[28] Svensson LG, Rodriguez ER (2005). Aortic organ disease epidemic, and why do balloons pop?. Circulation.

[29] Frydrychowicz A, Stalder AF, Russe MF, Bock J, Bauer S, Harloff A, Berger A, Langer M, Hennig J, Markl M (2009). Three-dimensional analysis of segmental wall shear stress in the aorta by flow-sensitive four-dimensional-MRI. J Magn Reson Imaging.

[30] Kilner PJ, Yang GZ, Mohiaddin RH, Firmin DN, Longmore DB (1993). Helical and retrograde secondary flow patterns in the aortic arch studied by three-directional magnetic resonance velocity mapping. Circulation.

[31] Morbiducci U, Ponzini R, Rizzo G, Cadioli M, Esposito A, Montevecchi FM, Redaelli A (2011). Mechanistic insight into the physiological relevance of helical blood flow in the human aorta: An in vivo study. Biomech Model Mechanobiol.

[32] Van Ooij P, Garcia J, Potters WV, Malaisrie SC, Collins JD, Carr JC, Markl M, Barker AJ (2016). Age-related changes in aortic 3D blood flow velocities and wall shear stress: Implications for the identification of altered hemodynamics in patients with aortic valve disease. J Magn Reson Imaging.

[33] Lorenz R, Bock J, Barker AJ, von Knobelsdorff-Brenkenhoff F, Wallis W, Korvink JG, Bissell MM, Schulz-Menger J, Markl M (2014) 4D flow magnetic resonance imaging in bicuspid aortic valve disease demonstrates altered distribution of aortic blood flow helicity. Magn Reson Med. 10.1002/mrm.24802 10.1002/mrm.24802 PMC3778148 23716466

[34] Dyverfeldt P, Hope MD, Sigovan M, Wrenn J (2013). Reproducibility of quantitative analysis of aortic 4D flow data. J Cardiovasc Magn Reson.

[35] Sigovan M, Dyverfeldt P, Wrenn J, Tseng EE, Saloner D, Hope MD (2015). Extended 3D Approach for Quantification of Abnormal Ascending Aortic Flow. Magn Reson Imaging.

[36] van Ooij P, Markl M, Collins JD, Carr JC, Rigsby C, Bonow RO, Chris Malaisrie S, McCarthy PM, Fedak PWM, Barker AJ (2017). Aortic valve stenosis alters expression of regional aortic wall shear stress: New insights from a 4-dimensional flow magnetic resonance imaging study of 571 subjects. J Am Heart Assoc.

[37] Bürk J, Blanke P, Stankovic Z, Barker A, Russe M, Geiger J, Frydrychowicz A, Langer M, Markl M (2012). Evaluation of 3D blood flow patterns and wall shear stress in the normal and dilated thoracic aorta using flow-sensitive 4D CMR. J Cardiovasc Magn Reson.

[38] Naim WNWA, Ganesan PB, Sun Z, Liew YM, Qian Y, Lee CJ, Jansen S, Hashim SA, Lim E (2016). Prediction of thrombus formation using vortical structures presentation in Stanford type B aortic dissection: A preliminary study using CFD approach. Appl Math Model.

